# A Review of Glass-Ionomer Cements for Clinical Dentistry

**DOI:** 10.3390/jfb7030016

**Published:** 2016-06-28

**Authors:** Sharanbir K. Sidhu, John W. Nicholson

**Affiliations:** 1 Adult Oral Health, Institute of Dentistry, Queen Mary University of London, London E1 2AD, UK; s.k.sidhu@qmul.ac.uk; 2 Dental Physical Sciences, Institute of Dentistry, Queen Mary University of London, London E1 2AD, UK; 3 The Bluefield Centre for Biomaterials, London EC1N 8JY, UK

**Keywords:** glass-ionomer cement, fluoride release, bioactivity, clinical applications, resin-modified, glass carbomer

## Abstract

This article is an updated review of the published literature on glass-ionomer cements and covers their structure, properties and clinical uses within dentistry, with an emphasis on findings from the last five years or so. Glass-ionomers are shown to set by an acid-base reaction within 2–3 min and to form hard, reasonably strong materials with acceptable appearance. They release fluoride and are bioactive, so that they gradually develop a strong, durable interfacial ion-exchange layer at the interface with the tooth, which is responsible for their adhesion. Modified forms of glass-ionomers, namely resin-modified glass-ionomers and glass carbomer, are also described and their properties and applications covered. Physical properties of the resin-modified glass-ionomers are shown to be good, and comparable with those of conventional glass-ionomers, but biocompatibility is somewhat compromised by the presence of the resin component, 2 hydroxyethyl methacrylate. Properties of glass carbomer appear to be slightly inferior to those of the best modern conventional glass-ionomers, and there is not yet sufficient information to determine how their bioactivity compares, although they have been formulated to enhance this particular feature.

## 1. Introduction

Glass-ionomer cements belong to the class of materials known as acid-base cements. They are based on the product of reaction of weak polymeric acids with powdered glasses of basic character [1]. Setting occurs in concentrated solutions in water and the final structure contains a substantial amount of unreacted glass which acts as filler to reinforce the set cement.

The term “glass-ionomer” was applied to them in the earliest publication [2], but is not strictly correct. The proper name for them, according to the International Organization for Standardization, ISO, is “glass polyalkenoate cement” [3], but the term “glass-ionomer” (including the hyphen) is recognised as an acceptable trivial name [4], and is widely used within the dental profession.

## 2. Composition

There are three essential ingredients to a glass-ionomer cement, namely polymeric water-soluble acid, basic (ion-leachable) glass, and water [4]. These are commonly presented as an aqueous solution of polymeric acid and a finely divided glass powder, which are mixed by an appropriate method to form a viscous paste that sets rapidly. However, alternative formulations exist which range from both the acid and the glass being present in the powder, and pure water being added to cause setting, to formulations in which some of the acid is blended with the glass powder and the rest is present in a dilute solution in water. This solution is used as the liquid component in forming the paste for setting. The effect of these differences is not clear, because these formulations are proprietary, so that the exact amount of each component is not widely known. However, there appears to be no obvious effect on the final properties of presenting these materials with the components distributed differently between the powder and aqueous phases.

Glass-ionomer cements can be mixed using a spatula on a pad or glass block, so-called hand-mixing. The material can also be presented in a bespoke capsule, separated by a membrane. The membrane is broken immediately before mixing, and the capsule is vibrated rapidly in a specially designed auto-mixer. This mixes the cement after which the freshly-formed paste is extruded from the capsule and used for intra-oral application.

Where a single brand is available as both a hand-mixed and capsulated version, the two types of cement have to be formulated differently. A cement paste that sets in a satisfactory time when hand-mixed sets far too rapidly when subject to vibratory mixing. As a result, formulations for capsulation have to be less reactive than those for hand-mixing, and they rely on the accelerating effect of auto-mixing to give them satisfactory working and setting times.

## 3. Polymeric Acids

The polymers used in glass-ionomer cements are polyalkenoic acids, either homopolymer poly(acrylic acid) or the 2:1 copolymer of acrylic acid and maleic acid. Poly(vinyl phosphonic acid) has been studied as a potential cement former [5], but its practical use is restricted to a single brand, where it is used in a mixture with poly(acrylic acid) and effectively acts as a setting rate modifier [6].

There is confusion in the literature about which polymers are used in glass-ionomer cements. This is because early research studied a range of mono-, di- and tri-carboxylic acid monomers in polymers for cement formation, including itaconic and tricarballylic acid [7]. This has led some authors to assume that these substances must be used in practical cements. However, this is not the case, and commercial cements use either the homopolymer or copolymer of acrylic acid.

The polymer influences the properties of the glass-ionomer cement formed from them. High molecular weights increase the strength of the set cement, but solutions of high molecular weight polymers have high viscosities, making them difficult to mix. Molecular weights are therefore chosen to balance these competing effects. Optimum properties are said to be achieved with average molecular weights of 11,000 (number average) and 52,000 (mass average) [8]. These values give a polydispersity of 4.7 [8].

Cements prepared from homopolymers of acrylic acid show increases in compressive strength in the first 4–6 weeks. On the other hand, cements made from acrylic-maleic acid copolymers show an increase in compressive strength up to a point, but then there is a decline before an equilibrium value is reached. Compressive strength is not a fundamental property of materials, because compression causes a specimen to fracture in complex ways in directions approximately at right angles to the compressive force. However, these alterations in measured compressive strength indicate that the material continues to undergo slow changes over time. In particular, this reduction has been attribute to the higher crosslink density that develops within copolymer cements compared with cements based on acrylic acid homopolymer [9]. In clinical use, however, this difference between the homopolymer and copolymer cements does not seem important and there is no evidence that cements made from acrylic/maleic acid copolymer are less satisfactory in service.

## 4. Glasses

It is vital that glasses for ionomer cements should be basic, i.e., capable of reacting with an acid to form a salt. In principle, several different glass compositions can be prepared that fulfil this requirement but in practice, only alumino-silicate glasses, with fluoride and phosphate additions, are fully satisfactory. Commercial glasses for glass-ionomer cements are typically based on calcium compounds, with some extra sodium. There are also materials in which calcium has been substituted by strontium.

Ionomer glasses owe their basic character to the fact that both alumina and silica are used in their preparation. Glasses based on silica alone lack reactivity and also basicity, because their structure contains mainly SiO_4_ tetrahedra linked at the corners to form chains that carry no charge. When alumina is added, the aluminium is forced to adopt a similar 4-fold tetrahedral geometry to silicon, i.e. AlO_4_ tetrahedra. As aluminium carries a formal 3+ charge, it does not counteract the effect of the negatively changed oxygens as effectively as silicon, with its formal 4+ charge. To balance this, extra cations such as Na^+^ and Ca^2+^ (or Sr^2+^) have to be present. These create basic character, and make the glass susceptible to attack by acids.

Fluoride is also a vital component of the glasses used in glass-ionomer cements. Glasses containing fluoride were among the earliest reported when glass-ionomers were first described, and were either of the SiO_2_-Al_2_O_3_-CaF_2_ system or the more complex SiO_2_-Al_2_O_3_-P_2_O_5_-CaO-CaF_2_ system [10]. An example composition is shown in Table 1, for the glass known as G338, which is similar to several commercial ionomer glasses.

Practical ionomer glasses, including G338, are known to undergo at least partial phase separation as they cool [10]. This leads to regions of varying composition and typically to the occurrence of one phase that is more susceptible to acid attack than the others. In principle, this might be expected to alter the optical properties of the glass, and in turn the cement, but there have been no studies reported exploring this point.

Studies of ionomer glasses have been carried out using MAS-NMR spectroscopy and these have provided useful structural information about these materials. Aluminium has been shown to occur in both 4- and 5-co-ordination in various glasses [11,12], which confirms the effect of silica on the co-ordination state of aluminium [12]. Fluorine occurs in these glasses bound exclusively to aluminium [13].

The substitution of calcium with strontium in glasses of this type can be achieved by using the compounds SrO and SrF_2_ in the place of CaO and CaF_2_ in the glass-forming mixture [14]. Strontium has the effect of increasing radiopacity compared with calcium in these glasses without any adverse effect on the appearance of these cements. Fluoride release is enhanced from these cements, though the reason for this is not known.

## 5. Chelating Additives

Several possible compounds have been studied as rate-modifying additives at either 5% or 10% by mass in cements [15]. Two of them proved highly successful, namely (+)-tartaric acid and citric acid and of these (+)-tartaric acid was the more effective.

The reasons for this are not clear. It may have something to do with its ability to prevent the precipitation of aluminium salts, which it does by chelating Al^3+^ ions and keeping them in solution [16]. By this mechanism, it may prevent the premature formation of ionic crosslinks involving Al^3+^ [17]. Certainly this is consistent with the fact that the bands due to aluminium polyacrylate appear later when tartaric acid is present than when it is absent. The bands arising from the various possible metal carboxylates occur in distinct regions of the infrared spectrum, as shown in Table 2.

The overall effect of including (+)-tartaric acid in a glass-ionomer cement is that setting is delayed, so that the cement is easier to mix. It then sets sharply to give the finished, hardened material that can be completed within the tooth. As a consequence of the ability to promote these changes, (+)-tartaric acid is a very useful additive. However, its effectiveness varies between glasses, depending on their composition.

## 6. Setting of Glass-Ionomer Cements

Glass-ionomers set within 2–3 min from mixing by an acid-base reaction. The first step is a reaction with hydrated protons from the polyacid at basic sites on the surface of the glass particles. This results in the movement of ions such as Na^+^ and Ca^2+^ (or Sr^2+^) from the glass into the polyacid solution, followed quickly by Al^3+^ ions. These ions then interact with the polyacid molecules to form ionic crosslinks, and the insolubilised polysalt that forms becomes the rigid framework for the set cement. When this setting reaction occurs, all of the water becomes incorporated into the cement, and no phase separation occurs.

Setting of glass-ionomer cements has been studied by various spectroscopic techniques, including infrared, FTIR and ^13^C NMR spectroscopy. The overall reaction appears to take place in two steps in a diffusion-controlled process [18]. The first step is the formation of ionic crosslinks, as we have seen, and this is responsible for the immediate hardening process. Subsequently, there is a crosslinking process involving Al^3+^ ions that takes about 10 min to be clearly identified spectroscopically [19]. This second step is slow, and continues for approximately a day [20].

After this initial hardening, there are further reactions, which take place slowly and are together known as maturation. They are associated with various changes in the physical properties of the resulting glass-ionomer cement [1]. Strength typically increases, as does translucency. In addition, the proportion of tightly-bound water within the structure increases. The details of these processes are not known, and research continues on this question.

Some years ago, it was shown that that hard, insoluble cements could be formed by reaction of ionomer glasses with acetic acid. This is in spite of the fact that metal acetate salts are soluble in water [21]. It was also observed that these cements became progressively stronger in compression up to 3 months, though there were no discernible changes in the infrared spectra of the cements. This led to the conclusion that there was an inorganic setting reaction that complemented the neutralization reaction in the setting of these cements. Metal silicates were proposed as substances responsible for this setting [21], but subsequent work on what became called “pseudo-cements” (i.e., cements made from monomeric acids with ionomer glasses) showed that insoluble materials resulted only with phosphate glasses. In contrast, phosphate-free silicate glasses were shown not to undergo an equivalent setting reaction [22]. This suggested that the proposed inorganic network is phosphate-based.

## 7. The Role of Water

As mentioned, water is the third essential component of the glass-ionomer cement. Several roles have been identified for water [9]. It is the solvent for the polymeric acid, it allows the polymer to act as an acid by promoting proton release, it is the medium in which the setting reaction takes place, and lastly, it is a component of the set cement [9].

Incorporation of water with glass-ionomers is associated with increases in the translucency of the glass-ionomer cement. The proportion of tightly-bound water increases with time for the first month or so of the cement’s existence, and several possible sites have been proposed. Binding may occur partly by co-ordination to metal ions and partly by strong hydration of the polyanion molecules [9]. In addition, it may react with –Si–O–Si– units at the surface of the glass particles, leading to the formation of –Si–OH groups [23]. This has been confirmed by a few FTIR studies where the relevant region of the spectrum has been examined. These studies have shown the occurrence of changes consistent with a reduction in the proportion of –Si–O–Si– groups (as shown by decreases in intensity of the band at 1060 cm^−1^) and increase in peaks due to –Si–OH (silanol) (one at 950 cm^−1^ [24] and one in the region 3435–3445 cm^−1^ [8]).

The unbound water can be lost from the surface of a newly placed glass-ionomer cement. This causes an unsightly chalky appearance as microscopic cracks develop in the drying surface. To prevent this, it is important to protect the cement by covering it with an appropriate varnish or petroleum jelly [25].

Two types of varnish are available, namely simple solutions of polymer in solvent and light-curable low viscosity monomer. There is evidence that the light-curable varnishes give superior protection in preventing drying out [25], because the lack of solvent means that the film formed has no porosities in it through which water can still escape.

## 8. Properties of Glass-Ionomers

The physical properties of glass-ionomer cements are influenced by how the cement is prepared, including its powder:liquid ratio, the concentration of the polyacid, the particle size of the glass powder and the age of the specimens. Care is needed therefore in making generalisations about the properties of these materials. There is also the possibility that part of the success of glass-ionomers may arise because their performance is satisfactory even if they have not been properly mixed, or allowed to mature under ideal conditions.

The current ISO standard for glass-ionomers [3] gives minimum values for certain physical properties. These values, which are shown in Table 3, are the least acceptable for a material to be allowed onto the market, rather than typical for materials known to perform well clinically.

The only type of strength that the ISO standard deals with is compressive strength, but glass-ionomers also have reasonable flexural strength [1]. Their biaxial flexure [26] and their shear punch strengths [27] have also been determined. As expected for a composite material, they show the same trends as compressive strength, typically improving at higher powder:liquid ratios and high concentration of polyacid.

## 9. Fluoride Release

Fluoride release is considered one of the important advantages of glass-ionomer cements [1]. It can be sustained for very long periods of time [28], and shows a pattern of an initial rapid release (“early burst”), followed by a sustained, lower level diffusion-based release [29]. These processes follow the pattern described by the equation [30]:

(1) $${\left[F\right]}_{c}=({\left[F\right]}_{1}\times \surd t)/\left(t+t_{1/2}\right)\;+\beta \cdot \surd t$$

In this equation [*F*]_c_ is the cumulative fluoride release at time *t* seconds, [*F*]_1_ is the total fluoride available, *t* is the time and *t*_1/2_ is the time taken for fluoride release to drop by a half, the so-called half-life of the release process. The initial term, ([*F*]_1_ × √t)/(*t* + *t*_1/2_), represents the “early burst” phase, though it has been found to last for up to four weeks. The second term, β·√t, in this equation is the long-term diffusion part of the release process.

Fluoride release from glass-ionomers increases in acidic conditions [31]. In addition, these cements are able to counteract such acidity, increasing the pH of the external medium. This process has been termed buffering, and may be clinically beneficial because it may protect the tooth from further tooth decay [31].

Release of fluoride in acidic conditions occurs with complexation. This may involve either aluminium ions, which are released in greater amounts than under neutral conditions, or hydrogen ions. The former may result in species such as ${\text{AlF}}_{4}^{-}$ [32], while the latter may cause the formation of either the complex ${\text{HF}}_{2}^{-}$ or undissociated HF [33]. None of these possible fluoride species yields free fluoride ions, so they are not detectable with fluoride-ion selective electrodes. Consequently the fluoride must be decomplexed to produce free F^−^ ions by the addition of TISAB (Total Ionic Solubility Adjustment Buffer). This is a proprietary solution supplied by various manufacturers for the purpose of decomplexing fluoride and ensuring that all of the fluoride in a sample is present as free anions.

Hydroxyapatite has been shown to react with acidic storage media from glass-ionomer cements to take up fluoride, regardless of whether or not the fluoride is complexed with any other chemical species [34]. These findings suggest that the increased amount of fluoride releases by glass-ionomers in acid conditions will increase the amounts of fluoride delivered to the mineral phase of the tooth [34].

Fluoride release is generally considered to be clinically beneficial. However, there is not yet convincing evidence to support this. A continuous supply of low levels of fluoride to the dental hard tissues is known to be beneficial [35] with concentrations at the parts per million level being sufficient to inhibit dentine demineralization by a measurable amount [36]. Fluoride release may also reduce hypersensitivity of the hard tissues towards cold foods and beverages. These amounts of fluoride seem achievable from glass-ionomer cements [37], but they have not been demonstrated over the longer term in saliva. To date, release has been mainly studied in pure water, and when artificial saliva has been used, much lower release levels have been observed [38]. Because of this, the likely clinical release into saliva over the long term is not known.

Fluoride is also taken up by glass-ionomer cements, at least in the early stages of a cement’s existence. This was originally proposed by Walls [39] and early experiments where release from a cement stored in water was compared with that from a cement stored in fluoride solution confirmed the idea [40,41]. Even fluoride-free glass-ionomers exposed to fluoride were shown to become fluoride-releasing when treated in this way [42].

Direct measurement confirms that fluoride is taken up by these cements [43]. However, this ability was found to be almost completely lost on maturation, so that 1 month old specimens of Ketac Molar Quick (3M ESPE, St Paul, MN, USA) and Fuji IX Fast (GC, Tokyo, Japan) did not take up any measurable fluoride at all [43]. These results suggest that fluoride recharge declines with maturation and that it is more complicated than many reports suggest [44]. Reports may anyway have exaggerated its potential importance, because the high-fluoride conditions under which a glass-ionomer restoration might be recharged will also cause the adjacent tooth mineral to take up fluoride. There will thus be protection from caries regardless of any enhanced fluoride release from the cement.

## 10. Adhesion

The adhesion of glass-ionomers to the surface of the tooth is an important clinical advantage. Glass-ionomers are prepared from poly(acrylic acid) or related polymers, and this substance has been known to promote adhesion, because of the adhesion of the zinc polycarboxylate cement [9]. The advantage conferred by their adhesion was exploited many years ago, when glass-ionomers were proposed for the repair of cervical erosion lesions and as pit and fissure sealants [45].

Tensile bond strengths of glass-ionomers to untreated enamel and dentine are good [46]. Values on enamel vary between 2.6 to 9.6 MPa and values on dentine vary from 1.1 to 4.1 MPa. Bond strengths are typically higher to enamel than to dentine, which suggests that the bonding takes place to the mineral phase [47]. Bond strengths develop quickly, with about 80% of the final bond strength being achieved in 15 minutes, after which it increases for several days [47].

Adhesion takes place in a number of stages. First, application of the fresh cement paste allows proper wetting of the tooth surface to take place. This is driven by the hydrophilic nature of both the cement and the tooth surface. Adhesion then develops rapidly, due to the formation of hydrogen bonds between the free carboxyl groups of the cement and the bound water on the surface of the tooth [48]. These hydrogen bonds are slowly replaced by true ionic bonds formed between cations in the tooth and anionic functional groups in the cement. This results in the slow formation of an ion-exchange layer between the tooth and the cement [49]. There is also the possibility of strong bonds between carboxylate groups of the poly(acrylic acid) and the surface, as shown by infrared spectroscopy [50]. Collagen does not seem to be involved in the bonding at all [50].

In the clinic, the tooth surface is prepared for bonding by conditioning, a process that involves treating the freshly cut tooth surface with a solution of 37% aqueous poly(acrylic acid) acid for 10–20 s followed by rinsing [47]. This technique removes the smear layer and opens the dentinal tubules, and also partially demineralizes the tooth surface. This leads to the surface area being increased and allows micro-mechanical attachment to occur [51].

Overall, therefore, adhesion of glass-ionomer cements can be attributed to two inter-related phenomena, namely:

1. Micromechanical interlocking, caused by glass-ionomers being self-etching due to the polyacid component.
2. True chemical bonding. This involves ionic bonds being formed between the carboxylate groups on the polyacid molecules and calcium ions in the tooth surface [51]. This has been observed experimentally on hydroxyapatite [52] and also on enamel and dentine [53] using X-ray photoelectron spectroscopy, though experimental conditions for these studies involve high vacuum, so requiring that the surfaces must be more strongly desiccated than under clinical conditions.

**Figure 1:**
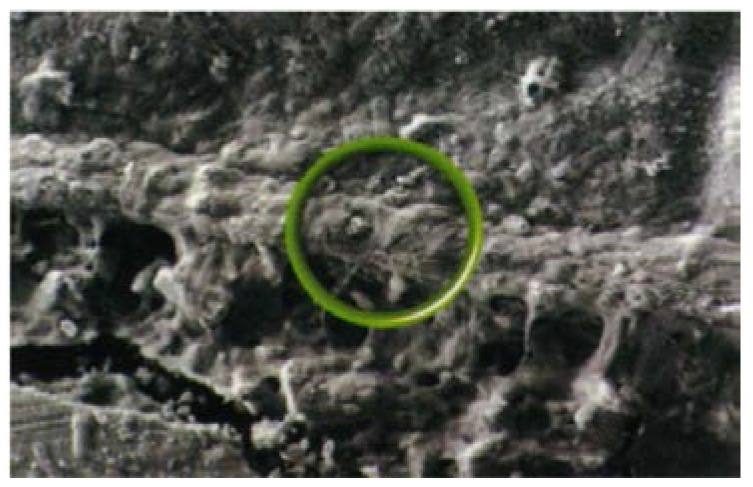
Interfacial ion-exchange layer formed between tooth surface (above) and glass-ionomer cement (below). The circle indicates part of the ion-exchange layer.

Over the longer term, a diffusion process occurs in which ions from the cement and ions from the tooth move into the interfacial zone and create an ion-exchange layer (Figure 1) [54]. This layer can be seen using scanning electron microscopy. The image in Figure 1 involved a strontium-based glass-ionomer cement, Fuji IX (GC, Tokyo, Japan), and analysis showed that the interfacial zone contained both strontium and calcium, indicating that this zone results from movement of ions from both the cement and the tooth. The resulting structure causes the cement and tooth to adhere strongly.

Studies show that failure of a glass-ionomer cement is usually cohesive, that is, it occurs within the cement, rather than at the interface. As a result, bonding values obtained in experiments are actually not measures of adhesive bond strength, but of the tensile strength of the cement. This strength is relatively low in freshly prepared specimens, but increases as cements mature. The consequence of this is that the quoted values in the literature are not the true measures of the adhesive bond strength of glass-ionomer cements.

Adhesion is important because it aids the retention of glass-ionomer cements within the tooth and also reduces or eliminates marginal leakage. This means that harmful micro-organisms are unable to enter the space under the restoration to promote decay.

## 11. Bioactivity

Glass-ionomer cements are naturally bioactive, partly because they release biologically active ions (fluoride, sodium, phosphate and silicate) into surrounding aqueous media at levels at which they are biologically beneficial [31]. In acidic conditions, these ions are released in larger quantities than in neutral conditions. In addition, calcium or strontium is also released, ions which occur in relatively insoluble compounds in neutral solutions. Under acidic conditions, glass-ionomers, too, will reduce the pH of the surrounding storage medium [31].

The ions released have a variety of biological roles. Phosphate occurs in saliva and in balance with the mineral phase of the tooth. Silicate can become incorporated into hydroxyapatite of the tooth without adversely affecting the crystal geometry [55], though whether it can do so with the mineral phase of teeth under clinical conditions is not clear. Calcium is an essential mineral element, with many biological uses. Within the mouth, it is the main counterion in hydroxyapatite, and in solution under mildly acidic conditions promotes remineralisation of the tooth.

As we have seen in connection with adhesion, the ability to exchange ions with the surroundings also applies to the solid tooth. With time, an ion-rich layer is formed which is very resistant to acid attack. Consequently, secondary caries is rarely observed around glass-ionomer restorations.

Glass-ionomers are also capable of taking up ions. In natural saliva, cements take up calcium and phosphate ions, and develop a much harder surface [56]. Related to this is the observation that, when used as fissure sealants, glass-ionomer cements form a substance deep within the fissure that has an increased content of calcium and phosphate and is much more resistant to cutting with a dental drill than the original tooth structure. This improved resistance to drilling, as well as the change in appearance, has been claimed to make the residual material resemble enamel [57].

## 12. Clinical Applications of Glass-Ionomer Cements

Glass-ionomers have various uses within dentistry. They are used as full restorative materials, especially in the primary dentition, and also as liners and bases, as fissure sealants and as bonding agents for orthodontic brackets. They can be classified into three types, depending on the intended clinical use, as follows:

Type I: Luting and bonding cements.

- For cementation of crowns, bridges, inlays, onlays and orthodontic appliances.
- Use relatively low powder:liquid ratio (1.5:1 to 3.8:1), leading to moderate strength only.
- Fast setting with good early resistance to water.
- Are radio-opaque.

Type II: Restorative cements.

There are two sub-divisions of Type II cements, depending on the importance of appearance.

For anterior repairs where appearance matters, Type II (i):

- Use high powder:liquid ratio (at least 3:1, and up to 6.8:1).
- Have a good colour match and translucency.
- Need protection from moisture for at least 24 hours with varnish or petroleum jelly.
- Are usually radio-opaque.

For use where appearance is not important (posterior restoration or repairs), Type II (ii):

- Also use high powder:liquid ratio (3:1 to 4:1).
- Fast set and early resistance to water uptake.
- Radio-opaque.

Type III: Lining or base cements

- Low powder:liquid ratio for liners (1.5:1) to allow good adaptation to the cavity walls.
- Higher powder:liquid ratio for bases (3:1 to 6.8:1), where the base acts as a dentine substitute in the “open sandwich” technique in association with a composite resin.
- Radio-opaque.

Much of the work reported on the clinical effectiveness of glass-ionomers has been anecdotal, and decisions on clinical applications have relied on the judgment and experience of clinicians. Recent attempts to review all of the published evidence have confirmed that glass-ionomers do have a measurable anti-caries effect. However, evidence to date is less clear about whether their fluoride release is beneficial in practice [58].

## 13. Fissure Sealants

Sealants of various types are placed in fissures of molars, either primary or permanent, to prevent caries developing by preventing the fissure from being colonised by plaque and pellicle [59]. Glass-ionomer was proposed for this application as long ago as 1974 [46].

Since this time, many studies have been carried out to compare the effectiveness of glass-ionomer cements and composite resin sealants. They have generally determined the relative retention rates, and mostly they have found that glass-ionomers are inferior in this respect [60]. However, when caries rate is considered, glass-ionomers prove to be as effective or superior to composite resins [61]. This may be due to retention of the cement deep within the fissure and also because of the anti-caries effects of the fluoride released by the cement [1].

Glass-ionomers have certain advantages over composites as fissure sealants, specifically that they are hydrophilic and dimensionally stable. Being hydrophilic they can absorb any fluid left at the bottom of the fissure and still adhere to the enamel. The dimensional stability allows the cement to retain its marginal adaptation and seal with the tooth. As a result, the risk of caries developing under the fissure sealing material is eliminated.

More recently, the development of high-viscosity glass-ionomers has provided a material that gives much better retention rates [61], and they now compare well with composite sealants. Their use in fissure sealing is therefore likely to continue well into the future.

## 14. Atraumatic Restorative Treatment (ART) Technique

Glass-ionomers are the materials used for tooth repair by the ART technique [62]. The technique has been developed under the aegis of the World Health Organisation with the aim of providing dental care in low- and middle-income countries. In these countries, caries is not properly managed and toothache is dealt with by extraction of the affected tooth. In addition, in these countries there are typically unreliable or non-existent electrical power supplies, which means that electrically driven drills and burs cannot be used routinely.

To address these problems, ART has been developed and introduced to various countries throughout the world. ART uses hand instruments to remove caries-affected dentine and enamel, after which high viscosity glass-ionomer cement is placed to repair the tooth [63]. Glass-ionomer cement is used because it is adhesive and can be used on tooth surfaces that have had only minimal preparation.

ART has been reported to be successful particularly for single-surface lesions. For example, in permanent teeth, after 2–3 years, Class I and Class V restorations had success rates of around 90% [64]. ART is used for children, who generally accept the treatment readily [62]. The technique has been successful in providing dental care for populations that would otherwise have minimal or non-existent care, and who would otherwise have had several teeth extracted [62].

## 15. Resin-Modified Glass-Ionomers

**Figure 2:**
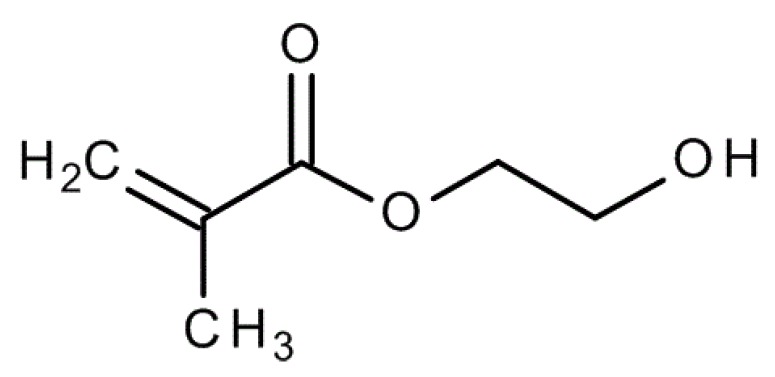
2-hydroxyethyl methacrylate (HEMA).

These materials were introduced to the dental profession in 1991 [65]. They contain the same essential components as conventional glass-ionomers (basic glass powder, water, polyacid), but also include a monomer component and associated initiator system. The monomer is typically 2-hydroxyethyl methacrylate, HEMA, (Figure 2) and the initiator is camphorquinone [65]. Resin-modified glass-ionomers set by the twin processes of neutralization (acid-base reaction) and addition polymerization, and the resulting material has a complicated structure based on the combined products of these two reactions [66]. Moreover, competition between these two network-forming reactions means that there is a sensitive balance between them [67]. This mixture of setting reactions may jeopardize the reliability of the set material, and as a consequence, close adherence to the manufacturer’s recommendations on the duration of the irradiation step is essential in order to produce material optimal properties [67].

Glasses employed in resin-modified glass-ionomers are the same as those used in conventional glass-ionomers. The acidic polymer may be the same, too, though in some materials, it is modified with side chains that end in unsaturated vinyl groups. These can become involved in the addition polymerization reaction and form covalent crosslinks between the polymer chains.

The physical properties of resin-modified glass-ionomers are comparable with those of conventional glass-ionomers [66]. They also release fluoride in a two-step process that is identical with that of conventional glass-ionomers in that there is an early wash-out phase followed by a sustained diffusion-based phase [29]. The kinetic equation describing this process is exactly the same as the one for conventional glass-ionomers [29,30].

Like conventional glass-ionomer cements, resin-modified glass-ionomers release small amounts of sodium, aluminium, phosphate and silicate under neutral conditions [68] Under acidic conditions, greater amounts are released and calcium (or strontium) is released as well [68]. Release of ions in acidic conditions is associated with a buffering effect, i.e., the pH of the storage medium gradually increases with increasing time of storage [69].

Biocompatibility of resin-modified glass-ionomers is markedly compromised compared with conventional glass-ionomers. This is due to the release of HEMA monomer, which is leached from resin-modified glass-ionomers in varying amounts mainly in the first 24 hours [70]. The amount released depends on the extent of light-curing that the cements have experienced [70]. HEMA is able to diffuse through human dentine [71] and is cytotoxic to the cells of the pulp [72].

HEMA from resin-modified glass-ionomers may also cause problems for dental personnel, as it is a contact allergen and is volatile, hence it is capable of being inhaled [73]. To ensure safe use of these materials, clinicians are recommended to use a well-ventilated workspace and to avoid inhalation of any vapour [74]. They are also advised to light-cure any unused remnants of material prior to disposal. Despite these concerns, there seem to be no case studies or reports in the literature of adverse reactions by patients or dental personnel to resin-modified glass-ionomers, though there is some anecdotal evidence of allergies developing in the latter group.

Resin-modified glass-ionomers have the same clinical applications as conventional glass-ionomers [75], though they are not recommended for the ART technique because of the need to use electrically-powered cure lamps. Thus, they are used in Class I, Class II and Class III restorations, all mainly in the primary dentition, Class V restorations and also as liners and bases [76]. Other uses include as fissure sealants [76] and as bonding agents for orthodontic brackets [77].

## 16. Glass Carbomer^®^

This is a novel commercial material of the glass-ionomer type, which has enhanced bioactivity compared with conventional glass-ionomer cement. It is manufactured by GCP Dental of the Netherlands. The name “glass carbomer” has been adopted in the scientific literature [77,78], which is unfortunate, because it is a brand name and the material is actually a type of glass-ionomer. It sets by an acid-base reaction between an aqueous polymeric acid and an ion-leachable basic glass, though it also contains substances that are not usually included in glass-ionomer formulations [79].

These components are as follows:

- A glass powder that has been washed by strong acid so that the surface layers of the particles are substantially depleted in calcium [80]. Hence, most of calcium ions lie well inside the particles towards the core.
- A silicone oil comprising a polydimethylsiloxane generally of linear structure, which contains hydroxyl groups. This allows the silicone oil to form hydrogen bonds with other components of the cement, so that it remains bound in the cement after setting.
- A bioactive component, which also behaves as a secondary filler. Solid state NMR spectroscopy has shown that this filler is actually hydroxyapatite [78] and it is included to promote the formation of enamel-like material at the interface with the tooth, as observed previously with conventional glass-ionomer fissure sealants.

The glass used in glass carbomer contains strontium, and also high amounts of silicon [78], as well as a small amount of calcium. It is relatively high in silicon compared with the glasses used in the well-established brands of conventional glass-ionomer Fuji IX and Ketac Molar, but it contains comparable amounts of aluminium, phosphorus and fluoride.

Due to the acid-washing process, the glass is fairly unreactive towards poly(acrylic acid) or acrylic/maleic acid copolymer. In addition, the silicone oil incorporated into the glass powder becomes adsorbed onto the surface of the glass, and this also interferes with the reaction with polyacid. As a result, the glass carbomer is easy to mix at high powder:liquid ratios, and only a little reaction occurs as these two components are blended.

Once the material is mixed, its sluggish setting reaction is speeded up by the application of a dental cure lamp for at least 20 s [79]. This is not to promote photo-polymerization, but because dental cure lamps give out heat. This increases the temperature of the cement, causing it to set in a reasonable time.

Glass carbomers contain high proportions of glass compared with conventional glass-ionomers, and also hydroxyapatite filler, so that the set glass carbomer would be very brittle. To overcome this, the silicone oil is added. It toughens the material, and remains bound within the material by hydrogen-bonding, as we have seen.

Studies of the setting reaction suggest that the setting of glass carbomer involves two parallel reactions, one involving the glass plus polyacid, the other hydroxyapatite plus polyacid. Both are acid-base reactions and result in an ionically crosslinked polyacid matrix containing embedded filler. In this case, however, the filler is not ion-depleted glass only, but also partially reacted hydroxyapatite. The resulting matrix is similar to that which occurs in a conventional glass-ionomer cement, though differs in that it also includes polydimethylsiloxane oil [80].

To date, there have been only preliminary reports on the use of glass carbomer clinically, and no long-term studies have been published. Consequently, the durability of the material in the mouths of patients is not yet known.

## 17. Conclusions

This review has shown from the published literature that glass-ionomer cements are versatile acid-base materials with a variety of uses in modern dentistry. They show a degree of bioactivity when set that causes them to develop an interfacial ion-exchange layer with the tooth, and this is responsible for the high durability of their adhesion to the tooth surface. They release fluoride for considerable periods of time, a feature which is generally considered to be beneficial, though evidence to support this is somewhat equivocal.

Modified forms of glass-ionomer are available, in the form of resin-modified glass-ionomers and glass carbomer. The former include a monomer and set in part by an addition polymerization, which augments the acid-base process and can be controlled using light activation. Physical properties of these materials are comparable with those of conventional glass-ionomers, but their biocompatibility is less good. Glass carbomer appears to be more brittle and less strong than the best modern conventional glass-ionomers. It releases fluoride, and the literature claims that it has been formulated with the aim of enhancing its bioactivity [78,80], though so far evidence to confirm this is lacking.

## Figures and Tables

**Table 1:** Composition of glass G338.

Component	% by mass
SiO_2_	24.9
Al_2_O_3_	14.2
AlF_3_	4.6
CaF_2_	12.8
NaAlF_6_	19.2
AlPO_4_	24.2

**Table 2:** Infrared absorption bands.

Salt	C–O Asymmetric Stretch (cm^−1^)	C–O Symmetric Stretch (cm^−1^)
Calcium polyacrylate	1550	1410
Aluminium polyacrylate	1559	1460
Calcium tartrate	1595	1385
Aluminium tartrate	1670	1410

**Table 3:** ISO requirements for clinical grade glass-ionomer cements.

Property	Luting Cement	Restorative Cement
Setting time/min	2.5–8	2–6
Compressive strength/MPa	70 (minimum)	100 (minimum)
Acid erosion (maximum)/mm h^−1^	**–**	0.05
Opacity, C_0.70_	**–**	0.35–0.90
Acid-soluble As/mg kg^−1^	2	2
Acid-soluble Pb/mg kg^−1^	100	100
